# Placental Aromatase Is Deficient in Placental Ischemia and Preeclampsia

**DOI:** 10.1371/journal.pone.0139682

**Published:** 2015-10-07

**Authors:** Alejandra Perez-Sepulveda, Lara J. Monteiro, Aneta Dobierzewska, Pedro P. España-Perrot, Pía Venegas-Araneda, Alejandra M. Guzmán-Rojas, María I. González, Macarena Palominos-Rivera, Carlos E. Irarrazabal, Horacio Figueroa-Diesel, Manuel Varas-Godoy, Sebastián E. Illanes

**Affiliations:** 1 Department of Obstetrics and Gynecology, Laboratory of Reproductive Biology, Faculty of Medicine, Universidad de Los Andes, Santiago, Chile; 2 Laboratory of Molecular Physiology, Faculty of Medicine, Universidad de Los Andes, Santiago, Chile; 3 Perinatal Unit, Clínica Dávila, Santiago, Chile; University Hospital Basel, SWITZERLAND

## Abstract

**Introduction:**

Preeclampsia is a maternal hypertensive disorder with uncertain etiology and a leading cause of maternal and fetal mortality worldwide, causing nearly 40% of premature births delivered before 35 weeks of gestation. The first stage of preeclampsia is characterized by reduction of utero-placental blood flow which is reflected in high blood pressure and proteinuria during the second half of pregnancy. In human placenta androgens derived from the maternal and fetal adrenal glands are converted into estrogens by the enzymatic action of placental aromatase. This implies that alterations in placental steroidogenesis and, subsequently, in the functionality or bioavailability of placental aromatase may be mechanistically involved in the pathophysiology of PE.

**Methods:**

Serum samples were collected at 32–36 weeks of gestation and placenta biopsies were collected at time of delivery from PE patients (n = 16) and pregnant controls (n = 32). The effect of oxygen tension on placental cells was assessed by incubation JEG–3 cells under 1% and 8% O_2_ for different time periods, Timed-mated, pregnant New Zealand white rabbits (n = 6) were used to establish an *in vivo* model of placental ischemia (achieved by ligature of uteroplacental vessels). Aromatase content and estrogens and androgens concentrations were measured.

**Results:**

The protein and mRNA content of placental aromatase significantly diminished in placentae obtained from preeclamptic patients compared to controls. Similarly, the circulating concentrations of 17-β-estradiol/testosterone and estrone/androstenedione were reduced in preeclamptic patients vs. controls. These data are consistent with a concomitant decrease in aromatase activity. Aromatase content was reduced in response to low oxygen tension in the choriocarcinoma JEG–3 cell line and in rabbit placentae in response to partial ligation of uterine spiral arteries, suggesting that reduced placental aromatase activity in preeclamptic patients may be associated with chronic placental ischemia and hypoxia later in gestation.

**Conclusions:**

Placental aromatase expression and functionality are diminished in pregnancies complicated by preeclampsia in comparison with healthy pregnant controls.

## Introduction

Preeclampsia (PE) is a pregnancy-specific disorder characterized by new-onset hypertension and proteinuria after 20 weeks of gestation [1,2]. It complicates in 5–7% of all pregnancies and is associated with an increased risk of maternal and fetal morbidity/mortality [2–5]. Moreover, a history of PE is associated with an increased risk of premature death remote from pregnancy due primarily to cardiovascular disease [1,4]. Although the precise etiology of PE remains unclear, it is now widely accepted that the pathophysiological process involves deficient trophoblast invasion of the maternal decidua and impaired remodeling of the maternal spiral arteries during the first trimester of pregnancy [5–8]. These circumstances result in deficient uteroplacental circulation, a poorly perfused and ischemic placenta and, hence, in the clinical syndrome of PE after 20 weeks of gestation [8–11]. In addition, the poorly perfused placenta is thought to synthesize and release increased amounts of vasoactive factors, disrupt the placental villous architecture, contributing to endothelial cell dysfunction [11,12]. As a result, an exaggerated maternal inflammatory response is generated by an imbalance in the concentrations of angiogenic [vascular endothelial growth factor (VEGF), placental growth factor (PlGF)] and anti-angiogenic factors [soluble endoglin (sEng), soluble vascular endothelial growth receptor–1 (sFlt1)], pro-inflammatory cytokines, and syncytiotrophoblast microparticles (STBMs) released into the maternal blood stream [11]. Moreover, chronic placental ischemia is also responsible for triggering oxidative stress and increase placental apoptosis, necrosis, and shedding of placental-produced debris in PE [13,14].

PE has been categorized into two distinct subtypes: early-onset PE, which develops before 34 weeks of gestation, and late-onset PE occurring at or after 34 weeks of gestation [11,15,16]. Both subtypes share overlapping presenting features but distinct biochemical markers, risk factors, clinical features and maternal and fetal outcomes.

During the last decade, various studies have measured the serum concentrations of androgens in PE [17–19]. Most of them have established that the circulating levels of testosterone and androstenedione are increased in PE compared to normotensive pregnancies, which led to suggestions that hyperandrogenism is a risk factor for the development of PE [18,20]. In contact, recent data demonstrate that estrogen, (17-β-estradiol) is diminished in PE [3]. In the human placenta, androgens derived from the maternal and fetal adrenal glands are converted into estrogens by the enzymatic action of placental aromatase. Specifically, androstenedione is converted into estrone, and testosterone into 17-β-estradiol [21,22]. This implies that alterations in placental steroidogenesis and, subsequently, in the functionality or bioavailability of placental aromatase are mechanistically involved in the pathophysiology of PE.

Aromatase is an enzyme complex constituted of 2 polypeptides: a flavoprotein, nicotinamide adenine dinucleotide phosphate-P450-reductase, expressed in all tissues and cell types of the body; and aromatase P450, expressed only in tissues that synthesize estrogens, including the gonads, adipose tissue, brain, and the placenta [22–24]. Aromatase is encoded by the *CYP19A* gene. Its transcription is tightly regulated through: tissue-specific promoter sequences [23]; the presence or absence of one or more single nucleotide polymorphisms (SNPs) [25,26]; and through hypoxia [27].

Since it has been suggested that impaired placental perfusion is responsible for the molecular events leading to the clinical manifestations of PE [8–10], we propose to investigate the expression and function of aromatase in normal and PE pregnancies in an *in vitro* model of hypoxia and in an *in vivo* model of placental ischemia.

## Materials and Methods

### Study design

A nested case-control study was conducted in the Obstetrics and Fetal Medicine Unit of the Hospital Parroquial de San Bernardo in Santiago, Chile. This study was approved by the Hospital Parroquial de San Bernardo and the Universidad de los Andes Ethics Committee, and written informed consent was obtained from all study subjects for collection of plasma and placental samples. Both cases and controls were selected from stored samples collected from a prospective cohort study performed between March 2008 and 2012 [28] that included women of Western European descent. Cases included women with singleton pregnancies who subsequently developed PE (n = 16) and controls included women with singleton pregnancies without chronic medical conditions or obstetric complications (n = 32). Serum samples were collected from all subjects at timed intervals during pregnancy (11–14 weeks, 22–24 weeks and 32–36 weeks) and stored at -80°C until further analysis. Placenta biopsies were collected from all subjects at time of delivery (described below). In this study, only serum samples from 32–36 weeks were used.

PE was defined as new-onset hypertension [(BP) blood pressure ≥140/90mmHg on two separate occasions at least 6h apart or BP ≥160/110mmHg)] and proteinuria (>300mg/24h) after 20 weeks of gestation in previously normotensive women.

### Tissue collection

Placental tissues were collected within 15 min of delivery according to standard placental sampling procedures [29]. Briefly, placental biopsies (~1cm^3^ spanning from the maternal to the fetal surface) were obtained from the placental cotyledon midway between the cord insertion and placental border, avoiding tissues from areas showing placental calcification or infarction. Tissues were washed twice in ice-cold normal saline to remove contaminating blood and stored. For protein assays, 2ml criovials were snap-frozen in liquid nitrogen until further analysis. For RNA analysis, samples were cut into smaller pieces (~0.1cm^3^) and placed into sterile DNase- and RNase-free 1.5ml microfuge tubes containing 1ml RNA*later* (Life Technologies, Grand Island, USA) and immediately placed at 4°C. After a period of 24h excess RNA*later* was removed and samples stored at -80°C until RNA isolation was performed.

### Measurement of estrogens and androgens concentrations in maternal circulation

Concentrations of aromatase metabolites in plasma samples obtained from cases and controls at 32–36 weeks of gestation were determined by radioimmunoassay (RIA). Testeosterone concentrations were measured by TESTO-RIA-CT kit, androstenedione concentrations by ANDROSTENEDIONERIA-CT kit and estrone concentrations by ESTRONE-RIA-CT. All kits were purchased from DIAsource (Louvain-La-Neuve, Belgium) and assays were performed according to manufacturer’s instructions. 17-β-estradiol concentrations were measured by Direct estradiol ^125^I Kit (Pantex, Santa Monica, USA) following the manufacturer’s instructions. Data were reported as absolute measurements as well as estrogen/androgen ratios.

### Cell Culture

The human JEG–3 choriocarcinoma cell line was a kind gift from Professor Gregory Rice (University of Queensland, Brisbane, Australia). This cell line is derived from metastatic lesions of choriocarcinoma, and has an extravillous trophoblast phenotype [30]. JEG–3 cells were cultured in phenol red-free RPMI 1640 medium (Life Technologies, USA), supplemented with 10% heat-inactivated fetal bovine serum (Thermo Scientific, Logan, USA), 1% (v/v) non-essential amino acids (Biological Industries, Israel) and 1mmol/L sodium pyruvate (Life Technologies, USA). The cell line was maintained at 37°C in a humidified incubator with 5% CO_2_. For hypoxia experiments, JEG–3 cells were seeded in 10 cm^2^ dishes and maintained in a humidified incubator (21% O_2_, 5% CO_2_, 37°C) for 16h prior to treatment. Cells were then incubated for different times (4, 8, 16 and 24h) either at 8% O_2_ or at 1% O_2_ in hypoxic C–474 chambers equipped with Pro-Ox 110 oxygen controlling regulator (Biospherix, Lacona, USA). The 0h time-point corresponded to cells left at 21% O_2_ for 16h before hypoxia exposure.

### *In vivo* model of placental ischemia

An established and validated *in vivo* animal model of placental ischemia was used [31]. Six pregnant New Zealand White rabbits (normal gestation period between 30–35 days) were provided by a certified breeder. For anesthetic induction, ketamine 35mg/Kg and xylazine 5mg/Kg were administrated intramuscularly. Briefly, the uterine spiral arteries supplying each placenta of one horn in day 25 pregnant rabbits were ligated to restrict blood flow to the placentas in that uterine horn by 50% (hypoxic horn). The spiral arteries of the placentae of the contralateral uterine horn were left intact and considered as controls (non-hypoxic horn). Animals were delivered by elective Cesarean section 5 days post-utero-placental restriction under the same anesthetic conditions and birth weight of individual pups recorded. Dams were sacrificed with pentobarbital 200mg/Kg via intravenous administration and pups were sacrificed by decapitation. Hypoxic (n = 10) and non-hypoxic (n = 17) placentae were collected. At least one hypoxic and one non-hypoxic placenta were collected from each of the 6 pregnant rabbits. The Universidad de los Andes Ethics Committee approved the animal experimentation of this study. Animal handling and all animal procedures were conducted in accordance to the same entity.

### Protein quantification

Cells were harvested by trypsinization and whole cell lysates prepared in RIPA lysis buffer [(50mmol/L Tris-HCl, pH 8.8; 150mmol/L NaCl; 0.5% (v/v) sodium deoxycholate; 0.1% SDS; 1% NP–40)] containing 1x complete protease inhibitor cocktail (Roche, Madison, USA). Human and rabbit placental tissues (~5mg) were homogenized using the Ultra-Turrax in RIPA lysis buffer including 1x complete protease inhibitor cocktail (Roche, USA). Both, the placental and cell lysates were incubated on ice for 20 min before centrifugation at 13,000 x g for 10 min at 4°C and the supernatant stored at -80°C. Total protein content was quantified using Pierce BCA Protein Assay Kit (Thermo Scientific, USA) and the NanoDrop 2000 spectrophotometer (Thermo Scientific, USA).

### Western blot analysis

Western blotting was performed on placental and whole-cell extracts. For placental samples, protein loading was normalized using Ponceau staining and densitometry. The aromatase (ab18995) rabbit polyclonal antibody (final dilution 1:2000) and hypoxia-inducible factor–1 alpha (HIF–1α) mouse monoclonal antibody (1A3) (final dilution 1:1000) were purchased from Abcam (Cambridge, USA), and the β-Tubulin (H–235) rabbit polyclonal antibody (final dilution 1:1000) was purchased from Santa Cruz Biotechnology (Santa Cruz, USA). Primary antibodies were detected using horseradish peroxidase-linked anti-rabbit or anti-mouse conjugates as appropriate (KPL, Gaithersburg, USA) and visualized using an ECL detecting system (Thermo Scientific, USA). The images derived from western blotting were analyzed by ImageJ (National Institutes of Health, Bethesda, MD, USA) software. Samples were normalized for protein loading by using β-Tubulin or Ponceau S staining.

### Quantitative real-time PCR

Total RNA was extracted from placental tissues and cell pellets with the MasterPure Complete RNA Purification Kit (Epicentre Biotechnologies, Madison, USA), followed by DNase I treatment. Complementary DNA generated by ImProm-II Reverse Transcription System (Promega, Madison, USA) was analysed by quantitative real-time PCR (qRT-PCR) in the Stratagene Mx3000P system (Agilent Technologies, Santa Clara, USA), using the Brilliant III Ultra Fast QPCR Master Mix (Agilent Technologies, USA). The following gene-specific primers and respective TaqMan probes were used for human aromatase: sense 5’-ATGAATCGGGCTATGTGGACGTGT–3’ and antisense 5’-TGGTTTGATGAGGAGAGCTTGCCA–3’ with probe 5’-TGAGGATCCCTTTGGACGAAAGTGCT–3’, and for rabbit aromatase: sense 5’-AAAGACGCAGGATTTCCACAGCAG–3’ and antisense 5’-TCAGCATCTCCAGCACACACTGAT–3’ with probe 5’-TTTGCTGAGAAACGTGGCGACCTGACAA–3’.

Transcript levels were determined by absolute quantification using the standard curve methodology. A purified RT-PCR aromatase product was sequentially titrated to create a standard curve and the unknown samples were therefore interpolated by the standard curve’s regression co-efficient.

### Statistical Analyses

Statistical analysis was performed using Mann-Whitney test for mRNA, hormone, and protein quantification experiments in human placentae and in the animal model and Student’s *t*-tests were used to assess differences in mRNA and protein content in the JEG–3 cell line. *P*≤0.05 was regarded as statistically significant.

## Results

### Clinical characteristics of the study population

The clinical characteristics of the study population at the time of enrollment are summarized in Table 1. At 11–14 weeks of gestation, those women who were destined to develop PE later in pregnancy already presented with significantly higher systolic and diastolic BP compared with controls. Although maternal age was not different between the two study groups, women who went on to develop PE already had a significantly higher weight and body mass index in the first trimester of pregnancy. Furthermore, women who developed PE presented with significantly lower gestational age at delivery and also delivered babies with significantly lower weight compared with controls.

**Table 1:** Clinical characteristics of controls and preeclampsia patients at different times of gestation.

	Normal Pregnancy (n = 32)	Preeclampsia (n = 16)	*p*-Value
**At 11–14 week visit**			
Systolic Pressure (mmHg)	107.3±1,9	121.4±3.6	0.0029**
Diastolic Pressure (mmHg)	64.7±1.4	72.3±3.8	0.0053*
Maternal weight (kg)	64.3±2.2	75.1±4.4	0.0263*
Body mass index (kg/m2)	25.4±0.9	30.4±1.7	0.0099*
**At 32–36 week visit**			
Systolic Pressure (mmHg)	111.2±1,7	125.1±2.9	0.0015**
Diastolic Pressure (mmHg)	67.7±1.5	75.4±2.5	0.0406*
**Time of delivery**			
Maternal Age (years)	25.5±1.2	29.9±1.9	0.0835
Gestation age at delivery (weeks)	39.3±0.2	36.6±1.0	<0.0001***
Birth Weight (g)	3523.8±70.2	3052.8±263.5	0.0301*
Newborn Gender (F:M)	19:13	7:9	0.3664

Values are given as Mean±SEM. Statistical significance was assessed using Mann Whitney test and Fisher's exact test.

*p≤0.05

**p≤0.01

***p≤0.001

### PE patients have reduced placental aromatase content compared to control pregnant women

**Fig 1:**
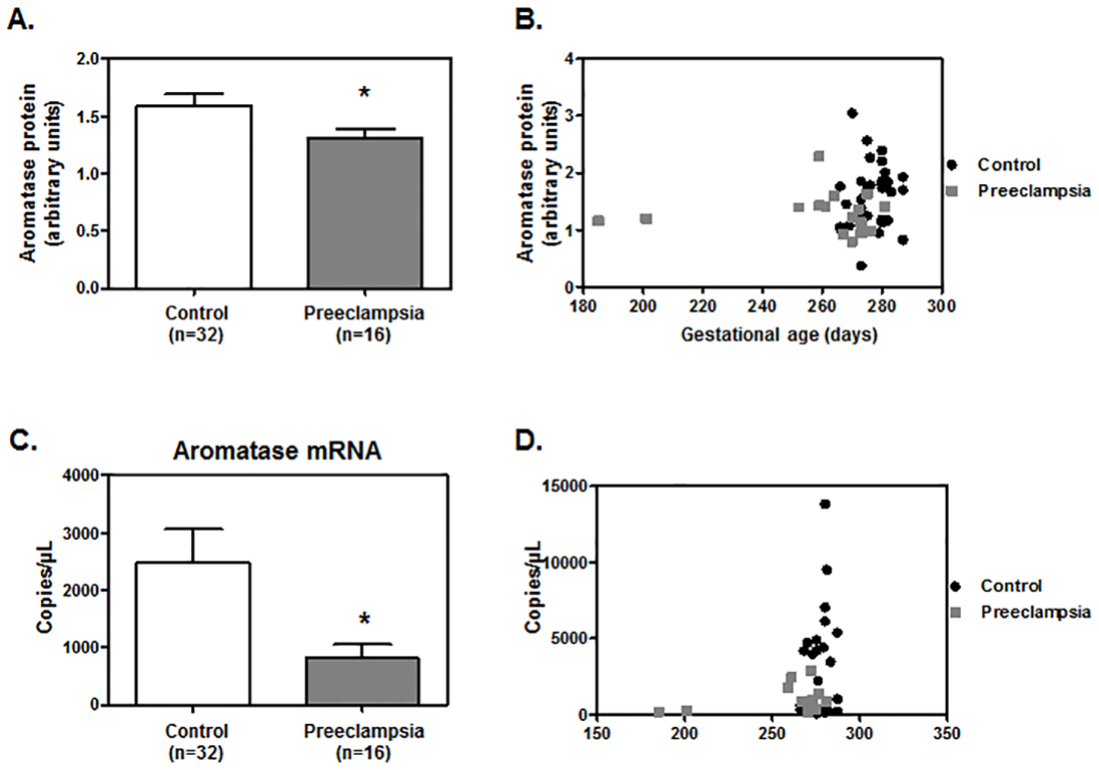
Placental aromatase (*CYP19A*) mRNA and protein levels are decreased in PE patients. Placental tissues from women with PE (n = 16) and control pregnant patients (n = 32) were collected **A**. and assessed for aromatase protein levels by western blotting. Aromatase protein band densiometry was normalized to an internal control. Each reported value derived from the ratio between arbitrary units obtained by the aromatase protein band and the respective Ponceau S staining. **C**. Placental aromatase mRNA levels were analyzed by qRT-PCR. Values are represented as mean±SEM. Statistical analysis was performed using Mann-Whitney test. *P≤0.05 significant. A linear regression model was applied to adjust **B**. aromatase protein and **D**. RNA expression to gestational age. Slops are not significantly different for gestational age and aromatase expression.

In order to address whether aromatase was dysregulated in human samples, the expression of placental aromatase was measured in placental tissues collected from women with and without PE (Table 1). Western blot analysis showed that PE placentas had significantly lower levels of aromatase protein compared with healthy controls (Fig 1A). Accordingly, qRT-PCR analysis also demonstrated that PE placentas presented significantly lower placental aromatase mRNA expression compared with controls (Fig 1C), indicating that placental aromatase is down regulated in PE. Furthermore, the linear regressions showing aromatase protein (Fig 1B) and RNA expression (Fig 1D) adjusted to gestational age demonstrate that for the same gestational age PE samples presented lower aromatase protein and RNA expression, respectively, than controls. These results indicate that the differences in expression of aromatase are not related to the difference in gestational age at delivery between PE and normal placentae.

### Aromatase function is compromised in PE patients

**Fig 2:**
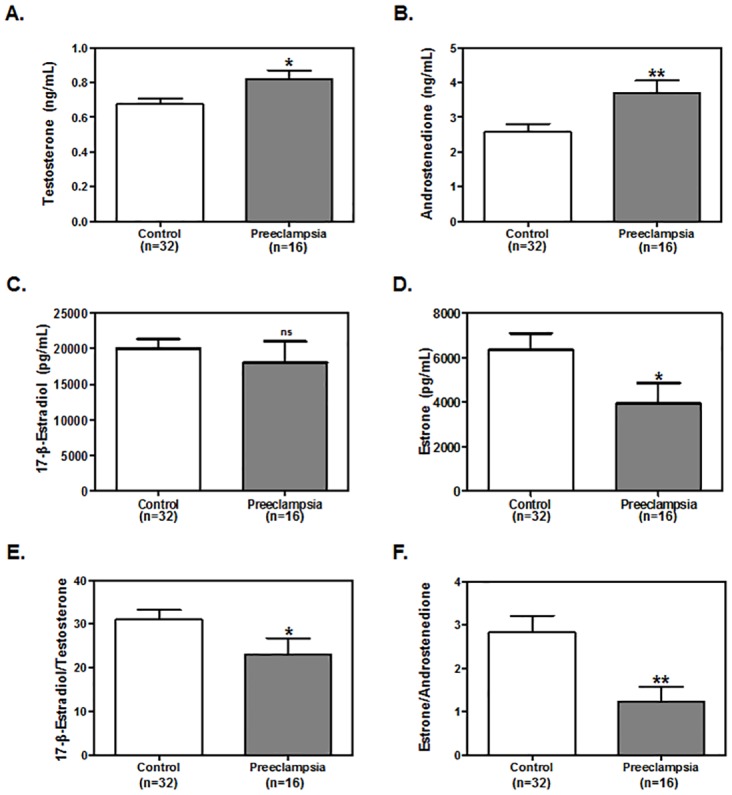
Aromatase metabolite levels are dysregulated in PE patients. Aromatase functionality was measured in PE and normotensive pregnancies. The levels of aromatase precursors and metabolites including, **A**. testosterone, **B**. androstenedione, **C**. 17-β-estradiol, and **D**. estrone were measured by RIA in maternal serum samples collected at 32–36 weeks of gestation. Also shown are **E**. 17-β-estradiol/testosterone and **F**. estrone/androstenedione ratios. Data are reported as mean±SEM from 32 controls and 16 PE patients. Statistical analysis was performed using Mann-Whitney test. *P≤0.05; **P≤0.01, significant; n.s., non-significant.

To assess aromatase function in PE and normotensive pregnancies, the concentrations of androstenedione, testosterone, estrone, and 17-β-estradiol were measured in 32–36 weeks maternal serum samples. Whereas circulating concentrations of 17-β-estradiol were not significantly different between the two groups (Fig 2C), concentrations of the 17-β-estradiol precursor, testosterone, were significantly higher in PE compared with normotensive pregnancies (Fig 2A). Moreover, circulating concentrations of estrone were significantly lower (Fig 2D) whereas concentrations of the estrone precursor, androstenedione, were significantly higher (Fig 2B) in PE patients compared with normotensive controls. Since the estrogens/androgens ratio is considered a reliable measure of aromatase functional status [3,32], these ratios were determined. Both 17-β-estradiol/testosterone (Fig 2E) ratio and estrone/androstenedione ratio (Fig 2F) were found to be significantly diminished in PE patients in comparison with normotensive controls. Taken together, these data suggest that aromatase function is dysregulated in women with PE and this dysregulation may lead to lower circulating concentrations of aromatase metabolites (estrone and 17-β-estradiol) and to the accumulation of aromatase precursor hormones (androstenedione and testosterone) in the maternal circulation.

### Aromatase is down regulated in response to hypoxia in trophoblast cell lines

**Fig 3:**
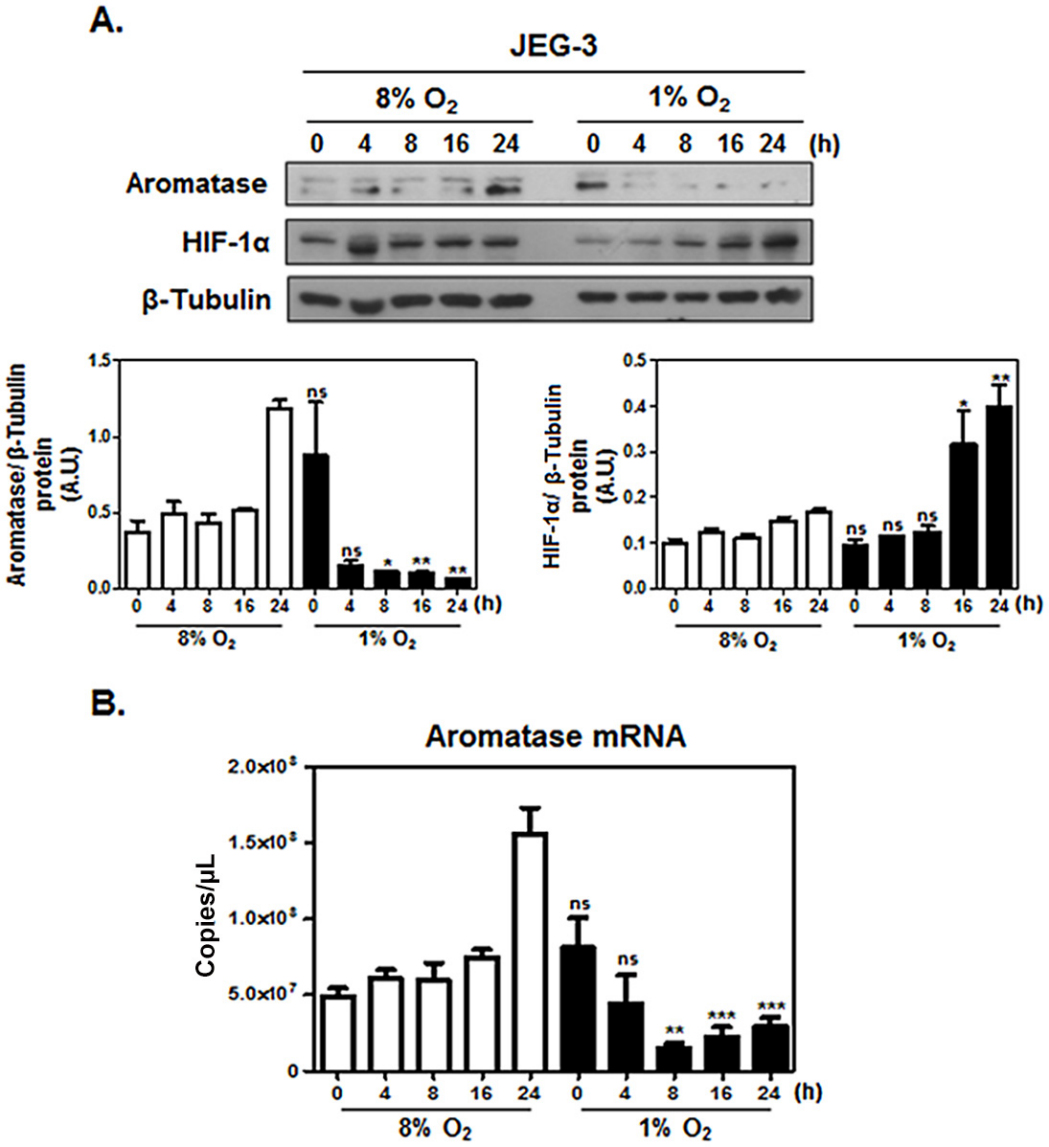
Aromatase is downregulated in JEG–3 cell line in response to hypoxia. JEG–3 cell line was exposed either to 8% or to 1% O_2_ in an hypoxic chamber for 0, 4, 8, 16 and 24 h. **A**. Cells were collected at indicated times and protein lysates analyzed by western blot to determine the protein expression levels of aromatase, HIF–1α and β-Tubulin. Upper panel shows representative western blots and lower panels show aromatase and HIF–1α proteins densitometry data normalized to β-Tubulin loading control from n = 3 experiments. Data are shown in arbitrary units (A.U.) ±SEM. **B**. Cells were collected at indicated times and analyzed by qRT–PCR to determine aromatase mRNA transcript levels. Statistical analyses were performed using Student’s *t*-test and compared with the correspondent time point in the control, 8% O_2_, cells. Columns are the mean of four independent experiments in duplicate; Data are reported as mean±SEM. *P≤0.05; **P≤0.01; *** *P*≤0.001, significant; n.s., non-significant.

In order to address whether aromatase was dysregulated in response to hypoxia, the choriocarcinoma cell line, JEG–3, was exposed to 8% O_2_ and 1% O_2_ for 4, 8, 16 and 24h and 0h are cells left at 21% O_2_ and regarded as control. The expression pattern of aromatase was investigated. Western blot analysis revealed that the expression of aromatase was significantly reduced following 8, 16 and 24 h exposure to 1% O_2_ compared to 8% O_2_ (Fig 3A). HIF–1α was included as a marker of hypoxia [2] and, as expected, was found to be upregulated under hypoxic conditions. qRT-PCR analysis confirmed that placental aromatase mRNA decreased following hypoxic treatment (Fig 3B), correlating with the changes seen at the protein level.

### Placental aromatase mRNA levels are reduced *in vivo* by induced placental ischemia

**Fig 4:**
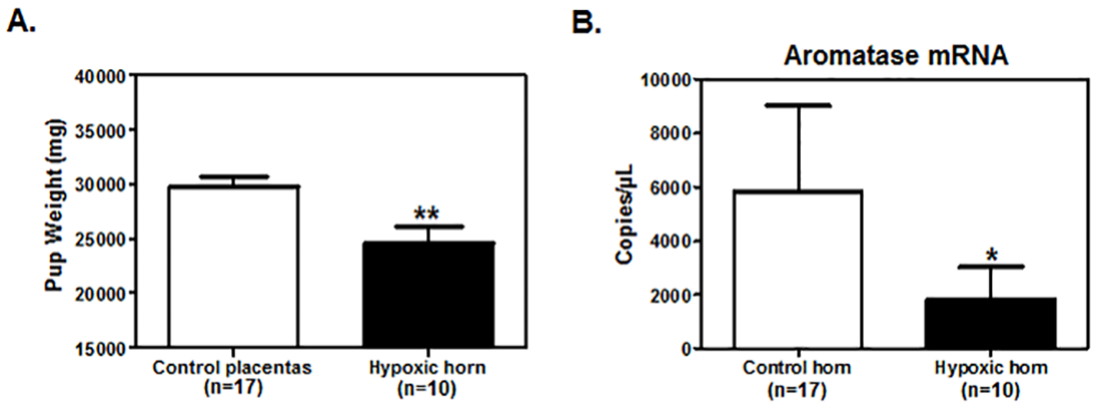
Effect of hypoxia on placental aromatase expression *in vivo*. **A**. Pups of timed-pregnant rabbits derived either from one uterine horn which placental spiral arteries had been ligated or from the non-ligated placentas on the contralateral horn (control horn, n = 17) were weighted **B**. and RNA was extracted from the correspondent placentas. Aromatase mRNA levels were analyzed by qRT-PCR in the control (n = 17) and hypoxic horn (n = 10). At least one hypoxic and one no-hypoxic placenta were collected from each of the 6 pregnant rabbits. Data are reported as mean±SEM. Statistical analysis was conducted using Mann-Whitney test. *P≤0.05; **P≤0.01, significant.

To further investigate the effect of hypoxia on placental aromatase expression, we used an established and validated pregnant rabbit model of placental ischemia [31]. As expected, pups from ligated placentae (hypoxic horn) were significantly smaller than those originated from the control placentas (control horn) (Fig 4A). Consistent with the *in vitro* data, placental aromatase mRNA expression was significantly lower in response to induced utero-placental ischemia versus non-intervened rabbit placentae (Fig 4B). These data suggest that preeclampsia associated with placental ischemia and late hypoxic events may be responsible for the diminished content of placental aromatase observed in preeclamptic patients.

## Discussion

Hypertensive disorders of the pregnancy remain a considerable obstetric problem worldwide with recent evidences suggesting the incidence of PE is increasing [2]. However, the pathophysiology of PE remains unknown. In this study, we demonstrate that placental aromatase expression and function is diminished in pregnancies complicated by PE and this impairment may be due to the chronic placental ischemia and hypoxia later in gestation that may lead to PE [33]. Indeed, we demonstrate that women who developed PE had significantly lower placental aromatase expression compared to healthy controls independently of gestational age. These results are in accordance with a previous study, which demonstrated an inverse correlation between concentrations of the estrogens, 17-β-estradiol and estrone, and the severity of PE [3]. These data suggest an impairment of steroidogenesis in women with PE and, therefore, a defect in aromatase expression since this enzyme is responsible for the conversion of androgens into estrogens. Consistent with results from a previous report [3], we identified lower concentrations of estrone in PE patients compared to controls. The concentrations of 17-β-estradiol in PE patients and controls, however, were not statistically different. We believe the use of the estradiol ^125^I-labelled ligand to detect 17-β-estradiol in the RIA assay may cross-reacted with other estradiols, such as 2-hydroxyestradiol, estriol and 17-α-estradiol, decreasing the specificity of the assay. It is relevant to mention that Hertig *et al*. measured the steroid profile of PE and control patients by GC/MS which, given the high homology of the molecular structure of these steroid groups, is a much more sensitive, precise and selective method compared to RIA. Furthermore, contrary to this study (that did not find altered concentrations of testosterone or androstenedione in PE [3]) we observed significantly higher androgen concentrations in the circulation of women with PE compared to normotensive women. This observation is supported by different reports that have identified androgen concentrations increased in women with PE, leading to the suggestion that hyperandrogenism may be a risk factor for the development of PE [18,20]. Nevertheless, there are no data available to correlate the elevated concentrations of androgen and the symptoms associated with PE. We further detected a significant decrease in 17-β-estradiol/testosterone concentrations and in the estrone/androstenedione ratios in women with PE, which accurately reflect the functional status of aromatase [3,32]. Although the evidence that plasma concentrations of these metabolites are altered in PE is compelling, the functional state of aromatase enzyme has not been directly assessed in women with PE. Since several recent studies [3,34,35], have found that circulating levels of 2-methoxyestradiol (2-ME), a natural metabolite of 17-β-estradiol, are downregulated in PE, we suggest that an alteration in the aromatase pathway could account for the lower concentrations of 2-ME in preeclamptic patientes. Since 2-ME is synthesized by COMT, decreased expression and/or activity of this enzyme could be responsible 2-ME low levels in PE. However, Palmer et al. has reported that placental COMT expression is not altered in severe PE compared to term or preterm normotensive pregnancies [36] and preliminary results of our group, also showed no significant changes in placental COMT protein expression between control and PE patients (unpublished data). Since the COMT enzyme did not seem to be related to the onset of PE, we hypothesized that the MHM cycle might be somehow altered in preeclamptic women, thus preventing the supply of sufficient methyl groups to sustain adequate concentrations of 2-ME. Nevertheless, quantification of functional polymorphisms present in methylenetetrahydrofolate reductase (MTHFR) and methionine synthase (MTR), key enzymes of the MHM cycle, revealed no significant differences in the frequency of SNPs between PE patients and control group [37]. Moreover, measurement of the plasma concentrations of SAM and SAH showed no differences in the amount of these metabolites between PE patients and normal pregnant women [37]. Overall, these pilot data indicate that neither the MHM cycle nor COMT enzyme are altered in women who subsequently develop PE and other pathway(s) may account for the lower levels of 2-ME in preeclamptic patients. Indeed, given that Hertig *et al* reported significantly lower concentrations of 17-β-estradiol in PE patients compared with controls [3], we believe there is a correlation between lower aromatase content and 17-β-estradiol concentrations and consequently the reduced 2-ME concentrations observed in PE. Nonetheless our results did not confirm this correlation, probably due to the lack of specificity of the method used to detect plasma concentrations of 17-β-estradiol. In an attempt to identify the mechanism(s) that contribute to the observed diminished aromatase content in PE patients, we hypothesized that placental aromatase is down regulated by placental ischemia. To mimic this phenomenon in an *in vitro* model, we exposed JEG–3 cells to different oxygen tensions. Indeed, the expression of placental aromatase was barely detected in response to 1% O_2_ in a JEG–3 cell line, which mimics the conditions of the placenta in the context of PE. Consistent with these data, a previous study reported that the expression, as well as the activity, of aromatase are diminished in primary trophoblast cells cultured under hypoxia (2% O_2_) and return to normal when normoxia (20% O_2_) is reestablished [27]. In our study, however, we considered 8% O_2_ as a normoxic environment. It has been postulated that during early pregnancy the oxygen concentration within the uterine surface is around 3–5% O_2_ (<20mmHg) [38]. Soon after the placenta establishes connections with maternal vasculature, oxygen tension increases to around 8.6% (~60mmHg) and remains at this level until birth [36,38]. Interestingly, we found aromatase content increased after 24h at 8% O_2_. A possible explanation for this major increase in aromatase expression is that 8% O_2_ tension may already be appropriate to decrease the activity of aromatase but not sufficient to affect its expression. Indeed, a study by Harada and colleagues has demonstrated that the inhibition of aromatase using two different pharmacologic inhibitors, are able to increase the levels of aromatase protein through stabilization and reduction of the protein turnover [39,40]. Therefore, in our model, cells exposed to 8% O_2_ may have lower aromatase enzyme activity which in turn could be triggering the re-synthesis of the protein producing its accumulation at 24h which coincides with the accumulation of the aromatase mRNA levels also seen at 24h. Additionally, we identified increased protein content of HIF–1α following 8-24h exposure to 1% O_2_. Interestingly, the placental environment of PE is also considered hypoxic and placentae from women with PE have also been reported to overexpress HIF–1α [41]. Consistent with the *in vitro* results, we also observed reduced placental aromatase expression in rabbit placentae exposed to induced placental ischemia. Whether aromatase expression is also down regulated by hypoxia in PE patients remains to be established. At this point we can only speculate that aromatase has a physiological role in the early stage of PE, considering the cell culture results, which is a closer model to first trimester trophoblasts due to its extravillous trophoblast phenotype, characteristic of the first trimester trophoblast [30]. It is relevant to mention, the role of placental hypoxia in the development of PE remains controversial. While some authors report that insufficient uteroplacental oxygenation in preeclampsia is responsible for the molecular events leading to the clinical manifestations of the disease [10,11,42], others suggest that placental hypoxia during early pregnancy does not play a role in the development of PE [16]. They maintain that PE is rather the result of a failure of villous trophoblast differentiation that leads to an abnormal release of trophoblast material (microparticles and other toxins) into the maternal circulation [16].

Another mechanism by which aromatase expression and activity may be altered is through the presence of SNPs within its promoter region [25,26]. Of particular interest are the SNPs in exon 1 of *CYP19A* (known as the placenta-specific aromatase regulator) that have been associated with specific changes in aromatase activity within the placenta as well as alterations in circulating concentrations of 17-β-estradiol [25]. We have measured three placental aromatase SNPs (rs4646, rs10046 and rs6493497) [43–45], each of which regulates the activity of the aromatase enzyme. We however, did not detect a significant difference between SNP frequencies amongst controls and PE patients (S1 Table). Additional studies are required to examine the association between SNPs in the *CYP19A* gene and PE in a larger population.

In conclusion, this study demonstrates that patients with PE have low placental aromatase content as well as low circulating concentrations of 17-β-estradiol/testosterone and estrone/androstenedione, indicating a loss of both aromatase expression and activity and this impairment may be due to chronic placental ischemia. Although these data are consistent with a role for aromatase in the pathogenesis of PE, a better understanding of the molecular and hormonal mechanisms responsible for the dysregulation of aromatase in PE is needed. Particularly, its effective preventive and/or early therapeutic interventions are to be developed, not only to reduce the associated morbidity and mortality during the pregnancy, but also the long-term sequelae associated with this disease.

## Supporting Information

S1 Table Genotype frequencies of the aromatase rs 4646, rs 10046 and rs 6493497 single nucleotide polymorphisms in controls and preeclamptic patients. (TIF)
