# Supplementary figures and images for: Placental Aromatase Is Deficient in Placental Ischemia and Preeclampsia
Source: PLoS One. 2015 Oct 7;10(10):e0139682. doi: 10.1371/journal.pone.0139682 (PMC4596497; doi:10.1371/journal.pone.0139682)

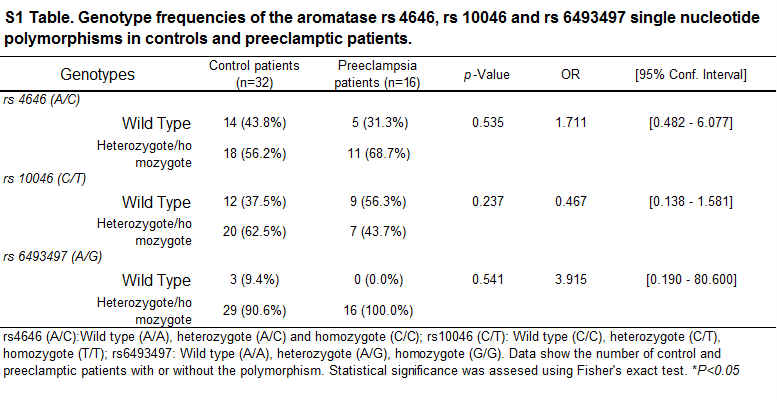

Supplement: S1 Table — (TIF) [file pone.0139682.s001.tif]
